# Assisted conception as a potential prognostic factor predicting insulin therapy in pregnancies complicated by gestational diabetes mellitus

**DOI:** 10.1186/s12958-019-0525-4

**Published:** 2019-10-27

**Authors:** Azam Kouhkan, Hamid Reza Baradaran, Roya Hosseini, Arezoo Arabipoor, Ashraf Moini, Reihaneh Pirjani, Alireza Khajavi, Mohammad E. Khamseh

**Affiliations:** 1 Reproductive Epidemiology Research Center, Royan Institute for Reproductive Biomedicine, ACECR, Tehran, Iran; 2 Department of Interdisciplinary Research in Diabetes, Obesity and Metabolism, Cell Science Research Center, Royan Institute for Stem Cell Biology and Technology, ACECR, Tehran, 19395-4644, Iran; 3 Endocrine Research Center, Institute of Endocrinology and Metabolism, Iran University of Medical Sciences (IUMS), Firouzeh St., South Vali- Asr Ave., Vali- Asr Sq, Tehran, Iran; 4 Ageing Clinical & Experimental Research Team, Institute of Applied Health Sciences, University of Aberdeen, Aberdeen, AB25 2ZD Scotland, UK; 5 Department of Andrology, Reproductive Biomedicine Research Center, Royan Institute for Reproductive Biomedicine, ACECR, Tehran, Iran; 6 Department of Endocrinology and Female Infertility, Reproductive Biomedicine Research Center, Royan Institute for Reproductive Biomedicine, ACECR, Tehran, Iran; 7 Department of Gynecology and Obstetrics, Arash Women’s Hospital, Tehran University of Medical Sciences, Tehran, Iran; 8 Breast Disease Research Center (BDRC), Tehran University of Medical Sciences, Tehran, Iran; 9 Student Research Committee, Faculty of Paramedical Sciences, Shahid Beheshti University of Medical Sciences, Tehran, Iran

**Keywords:** Gestational diabetes mellitus, Insulin, Prognostic factors, Assisted reproductive technology, Pregnancy

## Abstract

**Background:**

Advanced maternal age, family history of diabetes, pre-gestational obesity, increased level of HbA1c, history of gestational diabetes mellitus (GDM), and poor pregnancy consequences are considered risk factors for antenatal insulin requirement in women with GDM. However, the role of assisted reproductive technology (ART) in increasing the risk of insulin therapy in pregnancies complicated with GDM remained elusive. The current study aimed to determine the role of ART in predicting insulin therapy in GDM women and investigate the clinical and biochemical factors predicting the need for insulin therapy in pregnancies complicated with GDM.

**Methods:**

In this prospective cohort study, 236 Iranian women with GDM were diagnosed by one-step oral glucose tolerance test (OGTT) between October 2014 and June 2017. They were mainly assigned to two groups; the first group (*n* = 100) was designated as ART which was further subdivided into two subgroups as follows: 60 participants who received medical nutrition therapy (MNT) and 40 participants who received MNT plus insulin therapy (MNT-IT). The second group (*n* = 136) was labeled as the spontaneous conception (SC), consisting of 102 participants receiving MNT and 34 participants receiving MNT in combination with IT (MNT-IT). The demographic, clinical, and biochemical data were compared between groups. Multivariate logistic regression was performed to estimate prognostic factors for insulin therapy.

**Results:**

A higher rate of insulin therapy was observed in the ART group as compared with the SC group (40% vs. 25%; *P* < 0.001). Multivariate logistic regression demonstrated that maternal age ≥ 35 years [OR: 2.91, 95% CI: (1.28–6.62)], high serum FBS [1.10: (1.04–1.16)], HbA1c [1.91 (1.09–3.34)], and ART treatment [2.94: (1.24–6.96)] were independent risk factors for insulin therapy in GDM women.

**Conclusions:**

Apart from risk factors mentioned earlier, ART may be a possible prognostic factor for insulin therapy in pregnancies complicated with GDM.

## Introduction

Assisted reproductive technology (ART) is increasingly being practiced worldwide. Besides, gestational diabetes mellitus (GDM) has become more prevalent in obese women conceived via ART treatments [1]. Several lines of evidence demonstrate that both ART and GDM are associated with adverse pregnancy outcomes as compared to those with natural conception [2, 3]. Current reports indicate that a history of infertility can increase the risk of GDM independent of the known risk factors [4, 5]. Furthermore, a higher prevalence of GDM in women who became pregnant by ART was previously reported [5]. Therefore, GDM, as a marked co-morbidity, should be early diagnosed and managed appropriately.

As the pregnancy proceeds, insulin resistance is gradually increased, especially in the third trimester of the gestational period [6]. Obesity and insulin-resistance reduce the functionality of β-cells and induce inflammation thought to play key roles in the development of GDM [7]. In this circumstance, early management of GDM which is of immense importance includes medical nutrition therapy (MNT), self-blood glucose monitoring, physical activity, and regular consumption of medications to control hyperglycemia [8]. Approximately 20–60% of GDM women need pharmacological treatments to control their blood sugar [9]. Insulin is considered a safe and effective medication for women with GDM who failed to respond to medical nutrition therapy [8]. Adequate and accurate control of blood glucose can attenuate adverse maternal and perinatal outcomes [10]. However, factors predicting antenatal insulin requirement in women with GDM have not been fully understood. Some risk factors, such as advanced maternal age, family history of diabetes, pre-gestational obesity, high oral glucose tolerance test (OGTT) values, history of GDM or poor pregnancy consequences, and polycystic ovary syndrome (PCOS) have been previously addressed in the literature [11, 12].

However, the role of ART in increasing the risk of insulin therapy in women with GDM remained opaque. Hence, this study was designed to explore the role of ART in predicting insulin therapy in women with GDM. Moreover, we examined the predictive potential of clinical and biochemical parameters for insulin therapy in the management of women with GDM.

## Participants and methods

The present research was a prospective cohort study that included 236 Iranian singleton pregnant women (aged 20–40 years) with GDM who recruited between October 2014 and June 2017. All participants consisted of ART and spontaneous conception (SC) groups enrolled by simple sampling following GDM diagnosis. Medical records of Iranian GDM pregnant women were registered. The ART group included singleton pregnancies conceived following in-vitro fertilization /intra-cytoplasmic sperm injection (IVF/ICSI) or ICSI cycles that referred to the Department of Endocrinology and Female Infertility of the Royan Institute, Tehran, Iran. The protocol of infertility treatment in our institute was in agreement with the standard international guidelines.

The pregnant women with no history of infertility and/or infertility treatments were designated the SC group who referred to the Obstetrics and Gynecology Clinic of Arash Women’s Hospital which affiliated with Tehran University of Medical Science. Written informed consent was taken from all participants before the enrollment.

The diagnosis of GDM was made by a one-step OGTT at 24 and 28 weeks of gestation. The American Diabetes Association/International Association of the Diabetes and Pregnancy Study Groups (ADA/IAPDSG) criteria were considered to diagnose GDM [8]. The exclusion criteria were: (1) any systemic disorders, (2) pregravid diabetes or glucose intolerance, (3) previous insulin treatment, (4) consuming oral glucose-lowering drugs (metformin and glibenclamide), (5) vanishing embryos or selective fetal reduction, (6) history of polycystic ovarian syndrome (PCOS), and (7) twin pregnancies. The Institutional Review Board and Ethics committee of the Iran University of Medical Sciences and Royan institute approved the present study.

Clinical and demographic data were obtained from medical records and face-to-face interviews. In these two centers, the weight (without shoes with the least clothes) of women was measured by Seca scale, and the height was determined by a stadiometer. Pre-pregnancy body mass index (BMI) [pre-pregnancy weight (kg)/height (m) ^2^] was calculated according to the standard formula. Systolic and diastolic blood pressure was assessed by trained nurses with a mercury sphygmomanometer at 24–28 weeks of gestation. The mean systolic and diastolic blood pressure was recorded in duplicate. Venous blood samples were collected at 24 and 28 weeks of gestation for the determination of fasting blood sugar (FBS), hemoglobin A1c (HbA1c), insulin, a high-sensitivity C-reactive protein (hs-CRP), and interleukin*-*17 (IL-17), as well as the lipid profile, i.e., cholesterol, triglycerides (TG), high density lipoprotein (HDL), low density lipoprotein (LDL), and very-low-density lipoproteins (VLDL), after 8–12 h’ fast. The Homeostasis Model Assessment of insulin resistance (HOMA*-*IR) index was also calculated.

All GDM women referred to an endocrinologist and a dietician for blood sugar management, Medical nutrition therapy (MNT), and nutrition plan, and consultation. Medical nutrition therapy (MNT) was defined as the management of GDM with optimal diet (energy content, macronutrient distribution, its quality and amount) to achieve sufficient mother’s weight gain and fetal growth, as well as maintaining near-normoglycemia and avoiding the development of ketone bodies and hypoglycemia. The participants were asked to take three main meals with three snacks per day and perform self-monitoring of blood glucose (SMBG). After 2 weeks of MNT, all participants were visited again by an endocrinologist. Fasting and postprandial sugar (2 h after breakfast, lunch, and dinner) were evaluated. If FBS was lower than 95 mg/dl, the 1-h postprandial blood sugar level was < 140 mg/dl, and 2-h postprandial blood sugar level was < 120 mg/dl, MNT alone continued. Insulin therapy (IT) was initiated by an endocrinologist when medical nutrition therapy failed, and fasting/postprandial blood glucose levels were above the target at any time during pregnancy. Subcutaneous injections of the rapid-acting and/or long-acting insulin were prescribed according to the blood glucose patterns.

The ovarian stimulation protocols and follow-up process after standard IVF/ICSI procedures were described previously in details [13]. ART drugs, the protocol of controlled ovarian stimulation (COS) using standard GnRH agonists or antagonists, as well as the modes of ART (fresh or frozen embryo transfer cycles) were obtained from the medical registry of infertile women. Ovarian hyper-stimulation syndrome (OHSS) is characterized by the increased level of serum estradiol (> 4000 pg/ml) along with a large number (> 20 per ovary) of follicles on the day of human chorionic gonadotropin (hCG) administration. Infertile women who were at higher risk of developing OHSS, frozen embryo transfer was performed by the vitrification method.

Data related to the ART procedures, including menarche age, infertility duration, irregular menstrual cycle, infertility type (secondary vs. primary), history of ovarian hyperstimulation syndrome (OHSS) risk, ovarian stimulation protocol (standard GnRH agonists vs. GnRH antagonists), and the method of ART [fresh embryo transfer (fresh ET) vs. frozen ET)] were obtained from women receiving infertility treatments.

Other variables that were considered in the final analysis were as follows; maternal age, BMI, history of having a first-degree relative with diabetes, prior history of spontaneous abortion, and macrosomic baby, history of GDM, increased OGTT values (GTT-FBS and GTT-2 h), increased levels of HbA1c, mode of conception, and GDM treatment modalities (MNT /MNT- IT).

### Statistical analysis

In the current study, continuous variables were presented as the means and standard error of the mean (mean ± SEM) and categorical variables were expressed as the percentage. The chi-square test and independent T-tests were applied to compare variables between the two groups as indicated. The univariate logistic regression analysis was carried out to compare the characteristics of participants receiving either MNT-IT or MNT and select the variables for entering in the multivariate model, as well as determining significant predictive factors for insulin requirement in the study population. All statistical analyses were two-sided, and the *p*-value of < 0.05 was considered statistically significant. The analysis of the obtained values was performed by the Stata software version 12.

Based on previous studies [14–16], a sample size of 236 GDM women would be necessary to obtain a power of 80% with a significance level α = 5% to detect a relationship between the type of conception and the need for insulin therapy.

## Results

**Fig. 1:**
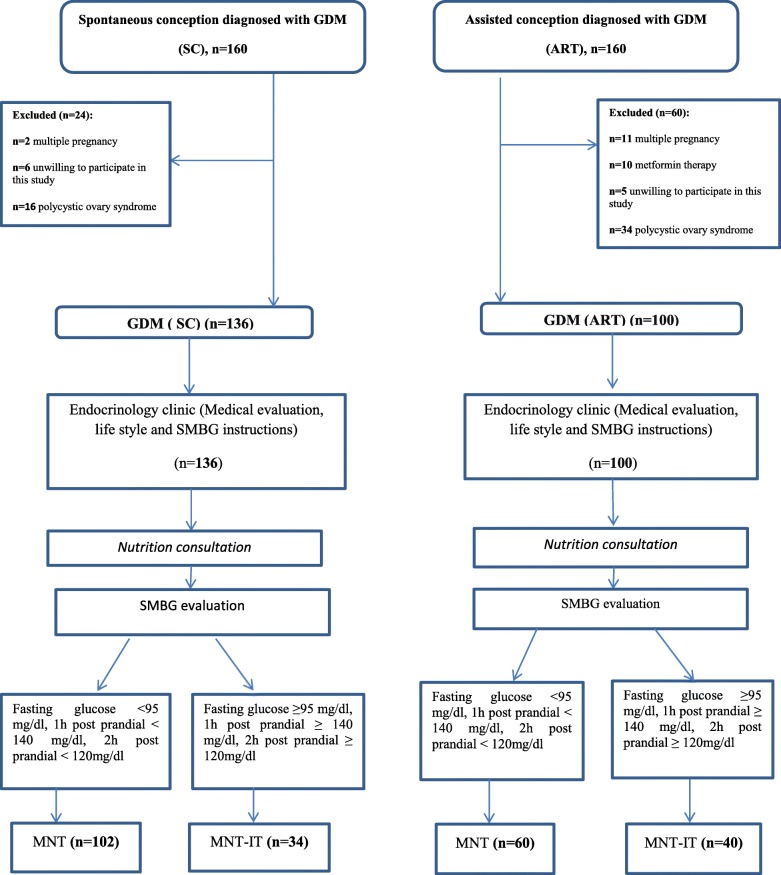
The Flow chart of the study population

In this study, 100 GDM women conceived via ART and 136 GDM women conceived via spontaneous conception were included. All participants were stratified based on the treatment modalities receiving during pregnancy [i.e., medical nutrition therapy (MNT) or medical nutrition therapy plus insulin therapy (MNT-IT)]. In the ART group, 60 subjects were in the MNT sub-group and 40 subjects in the MNT-IT subgroup. In the SC group, 102 subjects were assigned to the MNT sub-group and 34 subjects in the MNT-IT subgroup. Figure 1 shows a flow diagram of the categorization of participants. The results showed a higher rate of participants in the ART group who required insulin treatment compared with individuals in the SC group [40 (40%) vs. 34 (25%), respectively; *P* < 0.001].

The clinical and biochemical characteristics of women in both groups are summarized in Table 1. Based on our results, there was no significant difference between SC and ART groups in terms of the mean maternal age, systolic and diastolic blood pressure, and the number of individuals with a family history of diabetes, prior history of spontaneous abortion, and macrocosmic baby. However, there was a significant difference between the two groups concerning the parity, pre-pregnancy BMI, and history of GDM. Besides, most biochemical characteristics were not significantly different except for serum FBS and hs-CRP levels.

**Table 1:** Comparison of clinical and biochemical characteristics between spontaneous conception and ART participants

Variables	SC (*n* = 136)	ART (*n* = 100)	†*P*-value
Clinical			
Maternal age (Years, Mean ± SE)	31.57 ± 0.46	32.36 ± 0.52	0.261
Parity (*n* = 0), n (%)	56 (41.2)	88 (87.1)	0.001*
Family history of DM, (Yes, n %)	52 (38.2)	42 (41.6)	0.602
Systolic blood pressure (Mean ± SE)	107.07 ± 0.87	106.68 ± 0.99	0.769
Diastolic blood pressure (Mean ± SE)	68.59 ± 0.72	66.83 ± 0.76	0.098
Pre-pregnancy BMI (Mean ± SE)	25.89 ± 0.42	27.32 ± 0.40	0.018*
Prior history of spontaneous abortion, (Yes), n (%)	36 (29.1)	32 (31.7)	0.670
Prior history of GDM, (Yes), n (%)	18 (13.2)	1 (0.9)	0.001*
Prior history of macrosomia, (Yes), n (%)	6 (4.4)	1 (0.9)	0.124
Biochemical			
GTT-FBS (mg/dl)(Mean ± SE)	93.38 ± 0.87	93.13 ± 0.96	0.851
GTT-1 h(mg/dl)(Mean ± SE)	162.93 ± 4.11	158.22 ± 4.72	0.455
GTT-2 h(mg/dl)(Mean ± SE)	134.29 ± 3.71	133.26 ± 3.67	0.847
FBS (mg/dl)(Mean ± SE)	84.21 ± 1.05	88.23 ± 0.95	0.006*
HbA1c (%) (Mean ± SE)	4.98 ± 0.10	5.07 ± 0.63	0.297
TG (mg/dl)(Mean ± SE)	200.61 ± 6.13	193.42 ± 5.62	0.254
Cholesterol (mg/dl)(Mean ± SE)	219.91 ± 3.52	210.86 ± 4.43	0.107
HDL (mg/dl)(Mean ± SE)	64.17 ± 1.34	63.49 ± 1.26	0.715
LDL (mg/dl)(Mean ± SE)	116.13 ± 3.01	110.31 ± 4.01	0.238
VLDL (mg/dl)(Mean ± SE)	40.05 ± 1.24	37.19 ± 1.28	0.115
Hs-CRP (Mean ± SE)	4.69 ± 0.40	7.21 ± 0.83	0.005*
IL-17 (Mean ± SE)	1.19 ± 0.26	2.66 ± 0.95	0.161
Insulin (Mean ± SD)	13.57 ± 140	13.98 ± 0.96	0.821
HOMA-IR	2.90 ± 0.34	3.09 ± 0.23	0.672

*SC* spontaneous conception, *ART* Assisted reproductive technology, *IL-17* Interleukin- 17, *Hs-CRP* High-sensitivity C - reactive protein, The Homeostasis Model Assessment of *insulin resistance* (*HOMA*-*IR*) index = [Glucose] * [Insulin] / 405 (Glucose in mg/dl)

*P < 0.05 was considered statistically significant

† T-test compared the mean difference between the SC group and ART group

The clinical characteristics between the two groups were stratified based on treatment modalities that are displayed in Table 2. Higher rates of maternal age ≥ 35 years, multiparty, pre-pregnancy BMI ≥25 (kg/m^2^) and prior history of GDM were observed in the MNT-IT subgroup of the SC group; however, such increments were not statistically significant when compared with the MNT subgroup of the ART group. In the ART group, the MNT-IT subgroup had a higher rate of family history of diabetes compared with the MNT subgroup.

**Table 2:** Comparison of clinical parameters between parturients with spontaneous and ART conception stratified based on treatment modalities

Variables	SC (n = 136)				ART (*n* = 100)				OR3 (CI 5%) ^c^	*P*-value
	MNT (*n* = 102)	MNI-IT (*n* = 34)	OR1 (CI 95%) ^a^	*P*-value	MNT (*n* = 60)	MNT-IT (*n* = 40)	OR2 (CI 95%) ^b^	*P*-value		
Maternal age (Years, Mean ± SE)	30.64 ± 0.53	34.38 ± 0.73	1.17 (1.07–1.28)	0.001*	32.05 ± 0.77	32.65 ± 0.62	1.02 (0.95–1.10)	0.576	1.94 (1.10–3.41)	0.023*
Maternal age ≥ 35 years,n (%)	26 (25.5)	16 (47.0)	2.6 (1.16–5.82)	0.018*	19 (31.7)	15 (37.5)	1.29 (0.55–2.99)	0.546	1.87 (1.05–3.33)	0.032*
Parity (n = 0), n (%)	46 (45.1)	10 (29.4)	11.97 (0.86–4.54)	0.108	50 (83.3)	37 (92.5)	0.41 (0.10–1.58)	0.182	0.84 (0.47–1.47)	0.535
Family history of DM (Yes, n %)	35 (34.3)	17 (50.0)	1.91 (0.87–4.20)	0.103	20 (33.3)	22 (55.0)	2.44 (1.07–5.56)	0.033*	2.17(1.24–3.79)	0.007*
Systolic blood pressure (Mean ± SE)	106.37 ± 1.01	109.11 ± 0.72	1.03 (0.99–1.07)	0.176	107.25 ± 1.17	106.0 ± 1.82	0.99 (0.95–1.03)	0.542	1.01 (0.98–1.03)	0.424
Diastolic blood pressure (Mean ± SE)	68.22 ± 0.83	69.71 ± 1.42	1.02 (0.97–1.07)	0.367	66.83 ± 0.98	67.0 ± 1.25	1.00 (0.96–1.06)	0.915	1.0 (0.97–1.04)	0.574
Pre-pregnancy BMI (Mean ± SE)	25.44 ± 0.49	27.31 ± 0.81	1.08 (1.00–1.17)	0.059	27.22 ± 0.46	27.52 ± 0.76	1.01 (0.95–1.12)	0.726	1.94 (1.09–3.45)	0.022*
Pre-pregnancy BMI ≥25 (kg/m^2^), n (%)	49(49.1)	23 (71.9)	2.65 (1.12–6.31)	0.024*	43 (71.7)	29 (72.5)	1.04 (0.43–2.55)	0.928	1.92 (1.05–3.51)	0.034*
History of spontaneous abortion, n (%)	26 (26.0)	13 (38.2)	1.76 (0.77–4.02)	0.175	16 (26.7)	16 (40)	1.83 (0.78–4.30)	0.161	1.90 (1.12–3.23)	0.018*
History of GDM, (Yes, n %)	8 (7.8)	10 (29.4)	4.90 (1.74–13.74)	0.001*	1 (1.7)	0 (0)	–	1	2.66 (1.03–6.84)	0.043*
History of macrosomia, (Yes, n %)	4 (3.9)	2 (5.8)	1.53 (0.27–8.76)	0.630	1 (1.7)	0 (0)	0.87 (0.16–4.60)	0.872	0.66 (0.13–3.35)	0.619

*CI* Confidence Interval, *GDM* gestational diabetes mellitus, *SC* Spontaneous Conception, *ART* Assisted Reproductive Technology, *MNT* Medical Nutrition Therapy, *MNT-IT* Medical Nutrition Therapy plus Insulin Therapy

*P < 0.05 was considered statistically significant

^a^ OR1; Fitting GDM treatment modality as the outcome of the univariate regression models. The reference group was the MNT subgroup compared to the MNT-IT in SC group

^b^ OR2; Fitting GDM treatment modality as the outcome of the univariate regression models; The reference group was the MNT subgroup compared to the MNT-IT in ART group

^c^ OR3; Fitting GDM treatment modality as the outcome of the univariate regression models; The reference group was the MNT subgroup compared to the MNT-IT in the total population

The results of the univariate logistic regression analysis are presented in Table 2 and show the risk factors of insulin therapy in the study population. The parameters associated with insulin requirement in total population were as follows; maternal age ≥ 35 years, family history of diabetes mellitus (DM), pre-pregnancy BMI, previous history of spontaneous abortion, and prior history of GDM. However, there were no significant differences between MNT and MNT-IT subgroups of the ART group in terms of ART parameters (*P* > 0.05) (Table 3). None of the infertility parameters was associated with insulin therapy in this group.

**Table 3:** Infertility parameters of ART-conceived parturients stratified based on the treatment modalities

Variables	MNT (*n* = 60)	MNT-IT (*n* = 40)	*P*-value
Menarche age, years (Mean ± SE)	13.1 ± 0.2	13.6 ± 0.3	0.532
Infertility duration, years, (Mean ± SE)	6.2 ± 0.5	7.5 ± 0.8	0.117
Irregular menstrual cycle, n (%)	7 (11.7)	3 (7.5)	0.496
Infertility type (Secondary), n (%)	25 (41.7)	17 (42.5)	0.934
Prior OHSS risk, n (%)	23 (38.3)	17 (42.5)	0.677
COH protocol			
Standard long GnRH agonist, n (%)	44 (77.2)	35 (87.5)	0.199
ART mode			
Fresh ET, n (%)	28 (51.9)	23 (57.5)	0.587

*MNT* Medical Nutrition Therapy, *MNT-IT* Medical Nutrition Therapy plus Insulin Therapy, *Fresh ET* Fresh embryo transfer

*P < 0.05 was considered statistically significant

The biochemical values of the SC and ART participants were stratified according to the treatment modalities that are depicted in Table 4. The mean concentrations of FBS and HbA1c were significantly higher in the MNT-IT subgroup of the SC group compared with the MNT subgroup. Similar findings were observed in the MNT-IT subgroup of the ART group when compared with MNT subgroup. The higher levels of one- and two-hour glucose after OGTT were observed in the MNT-IT subgroup of the SC group in comparison with the MNT subgroup. However, such values were not significantly different between the MNT and MNT-IT subgroups of the ART population (P > 0.05). The mean concentrations of TG and VLDL were significantly higher in the MNT-IT subgroup of the ART group as compared with the MNT subgroup. The mean levels of the inflammatory markers were higher in the MNT-IT subgroup compared with the MNT subgroup of the ART group. In the total population, higher levels of GTT-FBS, GTT-1 h, GTT-2 h, FBS, and HbA1c, as well as hs-CRP, were observed in the MNT-IT subgroup compared with the MNT subgroup of the ART group.

**Table 4:** Comparison of biochemical parameters between parturients with spontaneous and ART conception stratified based on treatment modalities

Variables	SC (*n* = 136)			ART (*n* = 100)			*P*-value^c^
	MNT (*n* = 102)	MNI-IT (*n* = 34)	*P*-value ^a^	MNT (*n* = 60)	MNT-IT (*n* = 40)	*P*-value^b^	
GTT-FBS (mg/dl)(Mean ± SE)	91.69 ± 0.95	98.18 ± 1.66	0.001*	90.67 ± 1.20	96.92 ± 1.42	0.001*	0.002*
GTT-1 h (mg/dl)(Mean ± SE)	156.10 ± 4.46	183.90 ± 8.66	0.003*	159.14 ± 5.98	156.82 ± 7.98	0.813	0.014*
GTT-2 h (mg/dl)(Mean ± SE)	129.38 ± 4.19	147.40 ± 7.34	0.003*	131.0 ± 4.50	136.58 ± 6.39	0.464	0.046*
FBS (mg/dl) (Mean ± SE)	82.89 ± 1.21	88.03 ± 1.77	0.032*	85.98 ± 0.99	91.44 ± 1.77	0.004*	0.030*
HbA1c (%) (Mean ± SE)	4.92 ± 0.07	5.40 ± 0.12	0.005*	4.85 ± 0.10	5.16 ± 0.08	0.022*	0.004*
TG (mg/dl) (Mean ± SE)	201.12 ± 6.83	199.11 ± 13.71	0.887	175.31 ± 8.16	204.97 ± 9.69	0.026*	0.065
Cholesterol (mg/dl) (Mean ± SE)	221.33 ± 4.20	215.71 ± 6.36	0.491	202.09 ± 4.58	223.13 ± 7.97	0.019*	0.066
HDL (mg/dl) (Mean ± SE)	64.61 ± 1.56	62.85 ± 2.60	0.573	65.20 ± 1.74	60.81 ± 1.65	0.097	0.083
LDL (mg/dl) (Mean ± SE)	117.30 ± 3.89	112.52 ± 6.54	0.496	101.83 ± 4.35	121.42 ± 6.93	0.014*	0.105
VLDL (mg/dl) (Mean ± SE)	40.19 ± 1.36	39.59 ± 2.84	0.836	35.06 ± 1.66	40.90 ± 1.92	0.029*	0.089
hs-CRP (mg/l) (Mean ± SE)	4.60 ± 0.41	5.06 ± 1.16	0.651	6.45 ± 0.75	8.69 ± 1.95	0.049*	0.010*
IL-17(pg/mL) (Mean ± SE)	1.21 ± 0.27	0.70 ± 0.60	0.660	0.91 ± 0.19	5.52 ± 2.35	0.017^b^	0.061
Insulin (mU/L) (Mean ± SE)	12.90 ± 1.64	15.87 ± 2.58	0.375	13.26 ± 1.36	14.92 ± 1.29	0.411	0.191
HOMA-IR	2.76 ± 0.42	3.39 ± 0.49	0.443	2.86 ± 0.33	3.40 ± 0.31	0.255	0.202

*GDM* Gestational Diabetes Mellitus, *SC* Spontaneous Conception, *ART* Assisted Reproductive Technology, *MNT* Medical Nutrition Therapy, *MNT-IT* Medical Nutrition Therapy plus Insulin Therapy

The Homeostasis Model Assessment of *insulin resistance* (*HOMA*-*IR*) index = [Glucose] * [Insulin] / 405 (Glucose in mg/dl)

*P < 0.05 was considered significant

^a^ T-test compared the mean difference between MNT and MNT-IT subgroups in the SC group

^b^T-test compared the mean difference between MNT and MNT-IT subgroups in the ART group

^c^ T-test compared the mean difference between MNT and MNT-IT subgroups in total population

The analysis of multivariate logistic regression was conducted to determine the predictive factors linked with insulin therapy (Table 5). Factors identified as prognostic factors of insulin therapy were the age equal to or above 35 years old [OR: 2.91, 95% CI: (1.28–6.62)], increased levels of GTT- FBS [1.10: (1.04–1.16)], HbA1c [1.91 (1.09–3.34)], as well as ART treatment. The mode of conception (ART treatment) was identified as the independent prognostic factor for insulin requirement in GDM women after the adjustment (or controlling) of other confounding factors or covariates [OR: 2.94, 95% CI: (1.24–6.96)].

**Table 5:** The multivariate logistic regression analysis for the risk factors associated with insulin therapy

Model	OR (CI 95%)^a^
Age ≥ 35 yr. (Yes/No)	2.91 (1.28–6.62)†
Prior history of GDM (Yes/No)	3.22 (0.83–12.51)
GTT-FBS (mg/dl)	1.10 (1.04–1.16) †
GTT-2 h (mg/dl)	1.01 (0.99–1.02)
Family history of DM (Yes/No)	1.06 (0.45–2.45)
HbA_1_c (%)	1.91 (1.09–3.34)†
BMI ≥ 25 (kg/m^2^)	2.12 (0.84–5.37)
Mode of conception (ART/SC)	2.94 (1.24–6.96)†

Data are presented as odds ratios (95% confidence interval)

MNT-IT was compared to MNT: Reference group was MNT

†*P* < 0.05 was considered statistically significant

^a^ OR; Fitting GDM treatment modality as the outcome of the multivariate regression models;

## Discussion

The present study found that maternal age ≥ 35 years old, elevated fasting glucose, and increased levels of HbA1c were the independent risk factor for insulin therapy. Furthermore, infertility treatment using assisted reproductive technology may be a possible predictive factor for insulin therapy in women with GDM.

The prevalence of GDM in Iran ranges from 1.3 to 18.8% in different geographical regions [16]. A recent systematic review and meta-analysis revealed that GDM treatment reduces the risk of delivering infants with macrosomia (i.e., being large-for-gestational-age birth), shoulder dystocia, and gestational hypertension. Of note, GDM treatment causes no significant increase in the risk of small-for-gestational-age birth [17]. Regarding the global increment in gestational diabetes, determination of high-risk populations requiring insulin therapy is crucial. Lifestyle modification is the first-line therapy for the management of women with GDM. However, some GDM women require insulin therapy as they the change in their lifestyle would not be sufficient alone.

Assisted conception, a standard treatment for infertility, is growing worldwide. Several unknown and concomitant factors in women conceived via ART make them prone to develop complications during their pregnancy, such as gestational diabetes which influences the clinical practice. Recently, Chen et al. [18] observed that peripheral insulin sensitivity is reduced in IVF-conceived women. They also reported the alternation of glucose metabolism (impaired glucose tolerance) in IVF-conceived mice. On the basis of the current data, insulin requirement was significantly higher in the ART group compared with the SC group (40% vs. 25%). Previously, antenatal insulin requirement was reported in 10.8–52.8% of GDM women after spontaneous conception [5, 19].

Several studies investigated clinical and biochemical parameters predicting the need for insulin therapy in GDM women who spontaneously conceived [11, 12, 19–22]. Moreover, some studies suggested similar prognostic factors, including advanced age [11, 23], elevated fasting glucose [14, 15, 20, 23], elevated two-hour glucose [14], prior history of GDM [15], and HbA1c [12, 15, 20, 21] for insulin treatment. Conversely, some reports demonstrated that the elevated pre-pregnancy BMI [12, 14, 15, 23], family history of diabetes [12, 20], and elevated 1-h blood glucose after GTT [14, 15, 20] were potentially independent prognostic factors. More recently, Barens et al. indicated a prediction model for insulin therapy in GDM women. According to this model, seven significant independent prognostic factors have been introduced, namely maternal age > 30 years, pre-gravid obesity (BMI ≥30 kg/m^2^), prior history of GDM, FBS ≥ 5.3 mmol/l, HbA1c ≥ 5.5% at the initial diagnosis of GDM, early diagnosis of GDM (< 24 weeks of gestation), and family history of diabetes. They concluded that 85.7–93.1% of women had six to seven prognostic factors mentioned above, but 9.3–14% of women had no or one prognostic factor [11].

The present results revealed that age ≥ 35 years, elevated GTT-FBS, and HbA1c were the independent risk factors for insulin therapy. Interestingly, the current study found a new prognostic factor for insulin therapy in pregnant women with GDM. The risk of insulin requirement was 2.94 folds higher in the ART group compared with the SC group. However, little is known about the association between ART and the risk of GDM. Previous evidence showed that infertile women and ART population were susceptible to develop GDM [4, 13]; yet, the mechanism underlying ART-induced insulin resistance and insulin requirement is partially understood. We assessed the impact of some aspects of ART properties on antenatal insulin requirement and observed no significant difference between MNT and MNT-IT subgroups of the ART group with respect to the ART parameters. Several hypotheses may be proposed in this regard.

First, the experimental study showed ART-induced endothelial dysfunction and arterial hypertension, glucose intolerance, and insulin resistance [24]. Second, some ART characteristics may be in charge of insulin resistance and the need for insulin therapy, such as PCOS [25], the number of embryo transfer, and administration of GnRH agonist during the COS cycle in ART. Third, infertile women usually receive exogenous progesterone during the luteal phase and at the first trimester of pregnancy, which may be associated with gestational diabetes and insulin resistance. Similar mechanisms might be proposed for insulin requirement in patients who undergo ART treatment. Nunes et al. found that progesterone- particularly at pharmacological doses- increased the generation of reactive oxygen species (ROS), and it could be toxic to pancreatic β-cells as a result of oxidative stress [26].

Moreover, Wada et al. [27] showed the molecular mechanisms of progesterone involved in the pathogenesis of insulin resistance during pregnancy. They indicated that progesterone could induce insulin resistance by the inhibition of GLUT-4 translocation, a decrease in the expression of the insulin receptor substrate-1 (IRS-1), and the uptake of glucose by adipocytes. The degradation of IRS-1 is one of the primary mechanisms that could cause insulin resistance when exposed to pro-inflammatory cytokines [27]. Notably, our data showed the increased serum concentrations of inflammatory markers in the ART group. Furthermore, higher levels of inflammatory biomarkers were observed in the MNT-IT than those in the MNT group. Previous research also demonstrated the increased levels of hs-CRP in GDM women who underwent IVF-ET cycles [28].

Furthermore, recent evidence showed a correlation of the elevated hs-CRP [29] and pro-inflammatory cytokines [6, 7] with the development of GDM and insulin resistance. More recently, IL-13, as an inflammatory marker, was found to be associated with the conversion of normoglycemia into type 2 diabetes mellitus and the initiation of insulin therapy [30]. Hence, inflammatory biomarkers may have indirectly affected insulin requirement.

The current study demonstrates that assisted conception could be a prognostic factor for insulin requirement in GDM populations. However, there were some limitations in our study. We could not evaluate all aspects of ART characteristics, and only a limited number of inflammatory biomarkers were evaluated. Another limitation was the lower number of women requiring insulin treatment. Moreover, the current research was performed in Iranian GDM women. This may limit the generalizability of the findings to other racial and ethnic groups. It is necessary to study large populations with other racial/ethnic groups in the future. Additionally, it is possible that the underlying infertility, as opposed to ART, could account for the study findings and the present study was not capable of categorizing the effects of assisted conception (ART) and nature of infertility; hence, it is critical to consider this issue in future studies on infertile patients who undergo non-IVF treatment as a separate cohort.

In conclusion, our findings confirm that age ≥ 35 years old, elevated levels of GTT-FBS and HbA1c are regarded as the independent risk factors for insulin therapy in GDM population. Furthermore, assisted conception could be a predictive factor for insulin therapy in pregnancies complicated with GDM. However, this study is the first report in this field, and more studies are warranted to corroborate these results.

## Data Availability

The datasets used and/or analyzed during the current study are available upon the request from the corresponding authors.

## Abbreviations

ADA/IAPDSG: American diabetes association/ International association of diabetes and pregnancy study groups
ANOVA: Analysis of variance
aOR: Adjusted odds ratio
ART: Assisted reproductive technology
BSP: Blood sugar profile
COS: Controlled ovarian stimulation
GDM: Gestational diabetes mellitus
HbA1c: Hemoglobin A1c
HDL: High density lipoprotein
HOMA*-*IR: Homeostasis model assessment of insulin resistance
hs-CRP: High-sensitivity C-reactive protein
IL-17: Interleukin*-*17
IRS-1: Insulin receptor substrate-1
IT: Insulin therapy
IVF/ICSI: In-vitro fertilization/ Intra-cytoplasmic sperm injection
LDL: Low density lipoprotein
MNT: Medical nutrition therapy
OGTT: Oral glucose tolerance test
OHSS: Ovarian hyperstimulation syndrome
PCOS: Polycystic ovary syndrome
ROS: Reactive oxygen species
SC: Spontaneous conception
SMBG: Self-monitoring of blood glucose
TG: Triglycerides
VLDL: Very-low-density lipoproteins
